# Expression of Flavodiiron Proteins Flv2-Flv4 in Chloroplasts of Arabidopsis and Tobacco Plants Provides Multiple Stress Tolerance

**DOI:** 10.3390/ijms22031178

**Published:** 2021-01-25

**Authors:** Paula Vicino, Julieta Carrillo, Rodrigo Gómez, Fahimeh Shahinnia, Suresh Tula, Michael Melzer, Twan Rutten, Néstor Carrillo, Mohammad-Reza Hajirezaei, Anabella F. Lodeyro

**Affiliations:** 1 Instituto de Biología Molecular y Celular de Rosario (IBR-UNR/CONICET), Facultad de Ciencias Bioquímicas y Farmacéuticas, Universidad Nacional de Rosario (UNR), Rosario 2000, Argentina; vicino@ibr-conicet.gov.ar (P.V.); jcarrillo@ibr-conicet.gov.ar (J.C.); rlg.ggbio@gmail.com (R.G.); carrillo@ibr-conicet.gov.ar (N.C.); 2 Leibniz Institute of Plant Genetics and Crop Plant Research, OT Gatersleben, Corrensstrasse, D-06466 Stadt Seeland, Germany; fshahinnia@gmail.com (F.S.); tula@ipk-gatersleben.de (S.T.); melzer@ipk-gatersleben.de (M.M.); rutten@ipk-gatersleben.de (T.R.)

**Keywords:** flavodiiron 2-4, high light, drought, stress tolerance, *Nicotiana tabacum*, *Arabidopsis thaliana*, *Synechocystis*

## Abstract

With the notable exception of angiosperms, all phototrophs contain different sets of flavodiiron proteins that help to relieve the excess of excitation energy on the photosynthetic electron transport chain during adverse environmental conditions, presumably by reducing oxygen directly to water. Among them, the Flv2-Flv4 dimer is only found in β-cyanobacteria and induced by high light, supporting a role in stress protection. The possibility of a similar protective function in plants was assayed by expressing *Synechocystis* Flv2-Flv4 in chloroplasts of tobacco and Arabidopsis. Flv-expressing plants exhibited increased tolerance toward high irradiation, salinity, oxidants, and drought. Stress tolerance was reflected by better growth, preservation of photosynthetic activity, and membrane integrity. Metabolic profiling under drought showed enhanced accumulation of soluble sugars and amino acids in transgenic Arabidopsis and a remarkable shift of sucrose into starch, in line with metabolic responses of drought-tolerant genotypes. Our results indicate that the Flv2-Flv4 complex retains its stress protection activities when expressed in chloroplasts of angiosperm species by acting as an additional electron sink. The *flv2-flv4* genes constitute a novel biotechnological tool to generate plants with increased tolerance to agronomically relevant stress conditions that represent a significant productivity constraint.

## 1. Introduction

Oxygenic photosynthesis requires defined conditions of light and nutrient availability to operate at optimal efficiency, but phototrophic organisms, from cyanobacteria to higher plants, live and thrive in changing environments in which light intensity, temperature, and nutrient supply fluctuate widely during various timeframes. Under adverse environmental conditions or when CO_2_ is limiting (i.e., by stomatal closure), the reducing equivalents generated by the primary photochemical reactions of photosynthesis overcome the capacity of electron carriers to mobilize them to productive oxido-reductive pathways. As a consequence, the photosynthetic electron transport chain (PETC) becomes over-reduced and the excess of energy and reducing power can be delivered to O_2_, thus increasing the production of reactive oxygen species (ROS) such as the superoxide anion radical, peroxides, and singlet O_2_, which further compromise the functionality of the photosynthetic machinery [1,2]. Over the course of evolution, phototrophs have developed multiple responses to counteract the unwanted consequences of PETC over-reduction and ROS build-up. Most relevant among them are alternative electron transport (AET) pathways that dissipate the surplus of excitation energy and reducing equivalents, thus protecting photosynthesis from oxidative damage [3,4].

Dissipative systems involved in tolerance to environmental stresses include photosystem (PS) I-associated cyclic electron transport (CET); the Mehler–Asada pathway, also termed pseudo-CET, which involves O_2_ reduction with transient ROS formation, chlororespiration, and non-photochemical quenching (NPQ) that dissipates the excess of excitation energy as heat; and photorespiration in C3 plants [3,4,5]. Their relative contributions to stress tolerance depend on the type of organism and the nature and intensity of the challenge. Recently, a family of flavodiiron proteins (FDPs) was added to this suite of protective devices [6,7].

FDPs are present in prokaryotes, Archaea, some protozoa, algae, and gymnosperms, but not in angiosperms [8]. Those present in photosynthetic organisms are made up of three domains: an N-terminal β-lactamase-like domain containing a non-heme catalytic di-iron center, a flavodoxin-like domain with bound FMN, and a C-terminal NAD(P)H-flavin reductase domain with FAD/FMN and pyridine nucleotide binding sites [6,7,9]. Two FDPs named Flv1 and Flv3 are found in all phototrophs except angiosperms, whereas some β-cyanobacteria contain two additional isoforms, Flv2 and Flv4, and still other FDPs are present in heterocyst-forming cyanobacteria [7]. They have been reported to act as heterodimers, with the pairs Flv1-Flv3 and Flv2-Flv4 being the functional complexes [7]. Based on their redox potentials and analysis of cyanobacterial mutants, Flv1-Flv3 were shown to operate at the reducing side of PSI, presumably using ferredoxin (Fd) as an electron donor, whereas Flv2-Flv4 was initially proposed to subtract electrons from PSII, most likely from the quinone-B (Q_B_) primary acceptor [10]. Further research combining *Synechocystis* Flv-deficient mutants with inhibitors of the PETC revealed that Flv2-Flv4 could also accept electrons from PSI under conditions of ambient (low) CO_2_ availability or when overexpressed [11]. Both Flv dimers were shown to reduce O_2_ directly to water [12], therefore alleviating the electron pressure on the photosystems and eliminating dissolved O_2_ that can otherwise generate ROS by partial reduction or energy transfer. In the case of Flv2-Flv4, the O_2_ photoreduction pathway initiates downstream of PSI and is unrelated to Flv2-Flv4 interactions with PSII [11].

The Flv1-Flv3 proteins from cyanobacteria and their orthologues FlvA-FlvB from bryophytes have been extensively studied and shown to favor growth and reproduction under fluctuating light [13,14,15,16,17,18]. Moreover, they have been expressed in several angiosperms and their effects on photosynthesis, growth, and oxygen consumption reported in detail [19,20,21,22]. The results suggest that most of the protective roles played by Flv1-Flv3 in microorganisms and gymnosperms were taken over by other dissipative systems in angiosperms, most remarkably CET [19,21].

By comparison, knowledge on the function of Flv2-Flv4 lags way behind. The *flv2* and *flv4* genes of β-cyanobacteria are arranged in an operon that also contains an intervening open reading frame (termed Sll0218 in *Synechocystis*) in the order *flv4-sll0218-flv2* [7,11,23]. Expression of the operon is induced by high irradiation and low CO_2_ levels [24]. As in the case of Flv1-Flv3, Flv2 and Flv4 form a soluble complex that can bind to membranes in the presence of cations [23]. Sll0218 is a 19-kDa protein associated with a high molecular mass complex located in the thylakoid fraction. Deletion of the complete *Synechocystis* operon decreased the accumulation of PSII dimers [23,25], whereas its overexpression stimulated oxidation of the plastoquinone pool, inhibited the formation of singlet O_2_, and favored charge separation of PSII by increasing Q_B_ redox potential [10,25]. As a consequence, overexpressing cells were more resistant to photoinhibition of PSII and grew significantly faster than their wild-type (WT) counterparts under high light intensities [10].

The roles of the individual components of the *flv4-sll0218-flv2* operon were dissected by evaluating the phenotypes displayed by single *Synechocystis* mutants. The results suggested that the Flv2-Flv4 dimer supports PSII-dependent AET to a not yet identified electron acceptor. The Sll0218 protein, in turn, stabilized early intermediates of PSII repair and assembly, without affecting the function of existing centers [26]. Sll0218 also favored the productive attachment of phycobilisome antenna to the PSII core, thus optimizing light harvesting [23,26]. Expression of Flv2-Flv4 rescued PSII functionality in a full-deletion mutant in which Sll0218 was absent, but did not protect PSII from photoinhibition, confirming that the various members of the operon play different but complementary roles in PSII activity and stability [26].

The function of Sll0218 in energy transfer from the prokaryotic antennae to PSII reaction centers is most likely restricted to cyanobacteria, but the activity of Flv2-Flv4 as an electron sink might be operative in other photosynthetic organisms lacking phycobilisomes, such as plants. We therefore prepared tobacco (*Nicotiana tabacum*) and Arabidopsis (*Arabidopsis thaliana*) plants expressing Flv2-Flv4 in chloroplasts to investigate whether they can functionally engage in electron exchange reactions with the plant PETC and to characterize the stress tolerance of the resulting transformants. The results indicate that transgenic plants from the two species displayed increased tolerance to high irradiation and to other sources of stress such as salinity, redox-cycling oxidants, and water deprivation, as reflected by differential preservation of cell integrity, photosynthetic activity, and metabolic performance. The Flv2-Flv4 dimer thus played a critical role in relieving over-reduction of the PETC under stress conditions when expressed in plant chloroplasts.

## 2. Results

### 2.1. Preparation of Transgenic Plants Expressing Plastid-Targeted Synechocystis Flv2-Flv4

Simultaneous expression of Flv2 and Flv4 in tobacco and Arabidopsis was accomplished by cloning the corresponding *Synechocystis* genes in a single pCHF3 plasmid (see Section 4.1 in Materials and Methods). Transformation of the two model angiosperms was of interest to evaluate the generality of the potential effects assayed, and to take advantage of the different biochemical and genetic possibilities offered by these two experimental systems in further research.

**Figure 1:**
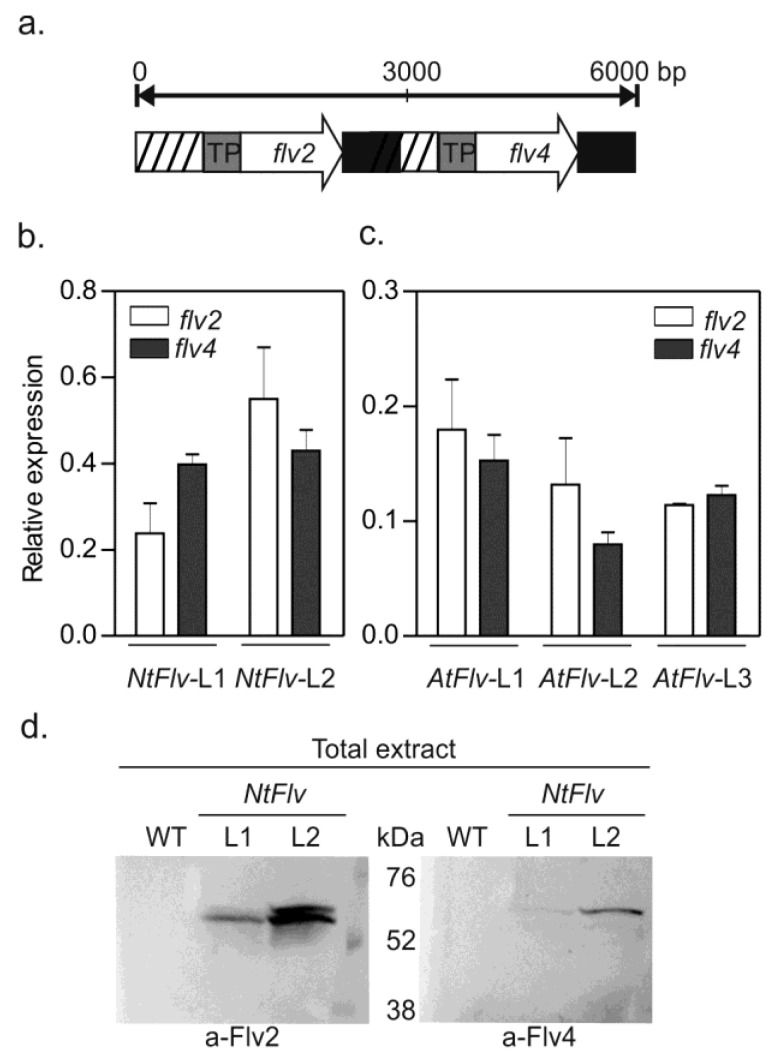
Expression of cyanobacterial Flv2-Flv4 proteins in tobacco and Arabidopsis plants. (**a**) Schematic representation of the T-DNA fragment of the pCHF3-*TP-flv2-TP-flv4* plasmid containing pea FNR TP coding sequences (grey boxes), *flv2*, *flv4* (white arrows), CaMV-35S promoters (striped boxes), and the Rubisco *rbcS-E9* terminator sequences (black boxes). Quantification of *flv* transcripts expressed in tobacco (**b**) and Arabidopsis (**c**) was carried out by qRT-PCR as described in Section 4.3 of Materials and Methods. Bars show means ± SE obtained from four plants for each independent line (biological replicates). (**d**) Flv expression in total leaf extracts of *NtFlv*-L1 and *NtFlv*-L2 tobacco lines was revealed by SDS-PAGE and immunoreaction with Flv2 and Flv4 antisera. Molecular weight standards are shown in kDa. Blots with the same exposure are shown. The uncropped scans of both Western blot images are presented in Figure S1a,b.

DNA sequences encoding the transit peptide (TP) of pea ferredoxin-NADP^+^ reductase (FNR) were fused in-frame to the 5′ end of the *flv* genes to allow chloroplast targeting (Figure 1a). Transgenic lines co-expressing Flv2-Flv4 were recovered for both tobacco (*NtFlv*) and Arabidopsis (*AtFlv*), as revealed by quantitative reverse-transcription PCR (qRT-PCR) amplification of leaf RNA (Figure 1b,c). Moreover, proteins migrating with a molecular mass similar to that of mature-sized Flv2 and Flv4 could be recognized in leaf extracts by reaction with specific antisera, as illustrated for tobacco leaves in Figure 1d. Analysis of band intensities indicated that the *NtFlv*-L2 line expressed significantly more Flv2 and Flv4 than its *NtFlv*-L1 sibling (Figure 1d and Supplementary Materials Figure S1a,b for complete gels).

Cellular localization of the introduced FDPs was investigated by fusing a C-terminal green fluorescent protein (GFP) tag to Flv2 and Flv4 (Figure S2a), and transiently expressing them in *Nicotiana benthamiana*. Figure S2b shows that GFP fluorescence co-localized with chlorophyll (*Chl*) auto-fluorescence in both cases, as revealed by confocal laser scanning microscopy (CLSM) of Flv-expressing leaves. GFP fluorescence was not detected in other cellular compartments (Figure S2b), confirming that the transgenic products were confined to chloroplasts in the transformed plants.

As indicated, the Flv2-Flv4 dimer of cyanobacteria can shift between the cytosol and the membrane fraction depending on the ionic composition of the medium [23]. We therefore analyzed the distribution of Flv2 in leaves of *NtFlv*-L1 and *NtFlv*-L2 plants using specific antisera. As shown in Figure S1a, the protein was detected in both the soluble and membrane fractions of tobacco leaf extracts, indicating that Sll0218 was not required for membrane association. Once again, Flv accumulation was significantly higher in the *NtFlv*-L2 line (Figure S1a). Flv4 was difficult to identify in the same extracts, possibly due to the lower quality of the antibody [26].

Transformants were able to set seeds, allowing for the selection of homozygous plants. Two independent lines of tobacco and three from Arabidopsis expressing both transgenes were used in further experiments. Expression of cyanobacterial Flv2-Flv4 did not introduce growth penalties to the transformed plants relative to their WT parentals when cultured under growth chamber conditions (see Section 4.2 in Materials and Methods). They actually showed a tendency to increase biomass accumulation in the conditions employed (Figure S3), although more systematic studies under different growth regimes would be required to properly substantiate this claim. The following sections describe examples of the increased tolerance displayed by Flv-expressing plants of the two species to an assorted suite of environmental stresses and conditions.

### 2.2. Flv2-Flv4 Protected Photosystem II from High Light Intensity in Tobacco Leaves

**Figure 2:**
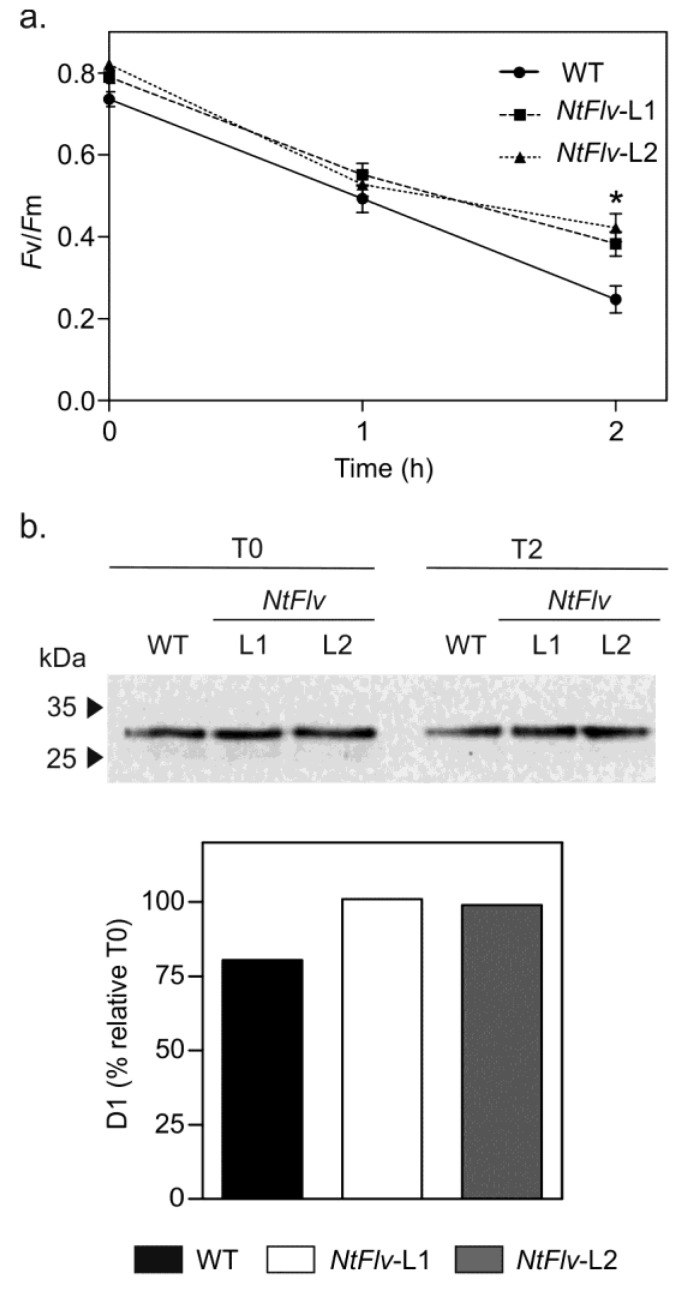
Flv2-Flv4 protected PSII from high light stress in tobacco leaves. Leaf discs from six-week-old tobacco plants were incubated with 3 mM lincomycin for 12 h in the dark and then exposed to 1200 µmol photons m^−2^ s^−1^. (**a**) *Fv*/*Fm* measured after 0, 1, and 2 h of treatment. *: the means differed significantly (*p* ≤ 0.05) from the performance of WT plants * using one-way ANOVA and Tukey’s multiple comparison tests. (**b**) Levels of D1 protein after 2 h of high light stress analyzed by immunoblot using D1 antibodies (upper panel) and quantified by densitometry of the corresponding bands (lower panel). Values are means ± SE of six biological replicates. The uncropped full scan of the western blot image is presented in Figure S4.

The activity of Flv2-Flv4 in cyanobacteria has been associated with the preservation of PSII functionality under conditions of excess irradiation [10,23,25]. We investigated whether a similar effect could be observed when the flavoproteins were expressed in plant chloroplasts. Tobacco leaf discs were exposed to 1200 μmol photons m^−2^ s^−1^ in the presence of lincomycin to prevent de novo protein synthesis, therefore allowing analysis of PSII degradation without the interference of repair processes [27,28]. The maximum efficiency of PSII *(Fv*/*Fm*), which is determined from chlorophyll (*Chl*) a fluorescence data and reflects PSII integrity [29], was employed to monitor photo-oxidative damage to the photosynthetic machinery. High irradiation resulted in *Fv*/*Fm* decline in WT and *NtFlv* lines, but significantly less in the transformants after 2 h of treatment (Figure 2a). Interestingly, the Flv-expressing plants displayed marginally increased *Fv*/*Fm* values even in the absence of light stress. These differences were not statistically significant at T0, but were amplified at longer exposure times (Figure 2a).

Light-dependent damage to PSII was confirmed by degradation of the D1 subunit from thylakoid membranes (Figure 2b and Figure S4). Flv2-Flv4 presence partially prevented D1 degradation, indicating that these flavoproteins can protect PSII from light stress in the absence of Sll0218 (Figure 2b and Figure S4).

**Figure 3:**
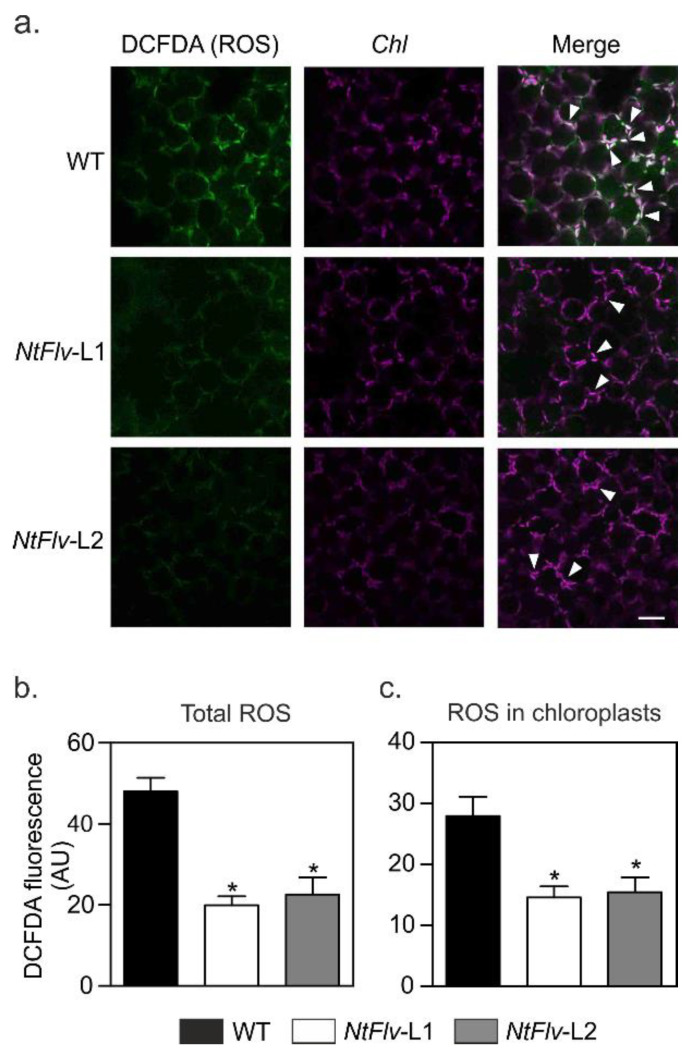
Flv2-Flv4 prevented chloroplast ROS build-up in tobacco leaves exposed to high light. Leaf discs from six-week-old plants were incubated with 3 mM lincomycin for 12 h in the dark, exposed to 1200 µmol photons m^−2^ s^−1^, and infiltrated with the ROS-sensitive probe DCFDA as described in *4.5* and *4.7* of Materials and Methods. (**a**) Fluorescent visualization of ROS formation in leaves by CLSM. ROS (green), *Chl* (magenta), and merge images are shown. Bar = 30 μm. The arrowheads show the merge of *Chl*- and ROS-derived signals in chloroplasts (see Figure S5 for amplification). ROS-derived fluorescence (**b**) and co-localization of the ROS-associated fluorescence with the *Chl* auto-fluorescence (**c**) were quantified on multiple image stacks using Fiji software. Fluorescence intensities are expressed in arbitrary units (AU). Data shown are means ± SE of four to six biological replicates. *: the means differed significantly (*p* ≤ 0.05) from the performance of WT plants using one-way ANOVA and Tukey’s multiple comparison tests.

ROS build-up is an early feature of many different environmental stresses, including high irradiation, and contributes to PSII inactivation and D1 degradation [2,30]. We therefore estimated ROS levels in tobacco leaves after high light exposure using the fluorescent probe 2′,7′-dichlorofluorescein diacetate (DCFDA), which is able to react with an assortment of ROS, including peroxides and singlet oxygen [31,32]. Figure 3a shows typical images obtained by CLSM, whereas Figure 3b,c provides quantitative fluorescence data determined by image analysis and averaged through several experiments. Most of the label was associated with chloroplasts, co-localizing with *Chl* auto-fluorescence (Figure 3a and Figure S5). The presence of plastid-targeted Flv2-Flv4 resulted in significantly less DCFDA fluorescence compared to WT counterparts (Figure 3). It is worth noting, within this context, that over-expression of the *flv2*/*4* operon correlated with reduced production of singlet O_2_ in *Synechocystis* cells [10].

### 2.3. Flv2-Flv4 Provided Increased Tolerance to Salt and Oxidative Stress in Tobacco and Arabidopsis

**Figure 4:**
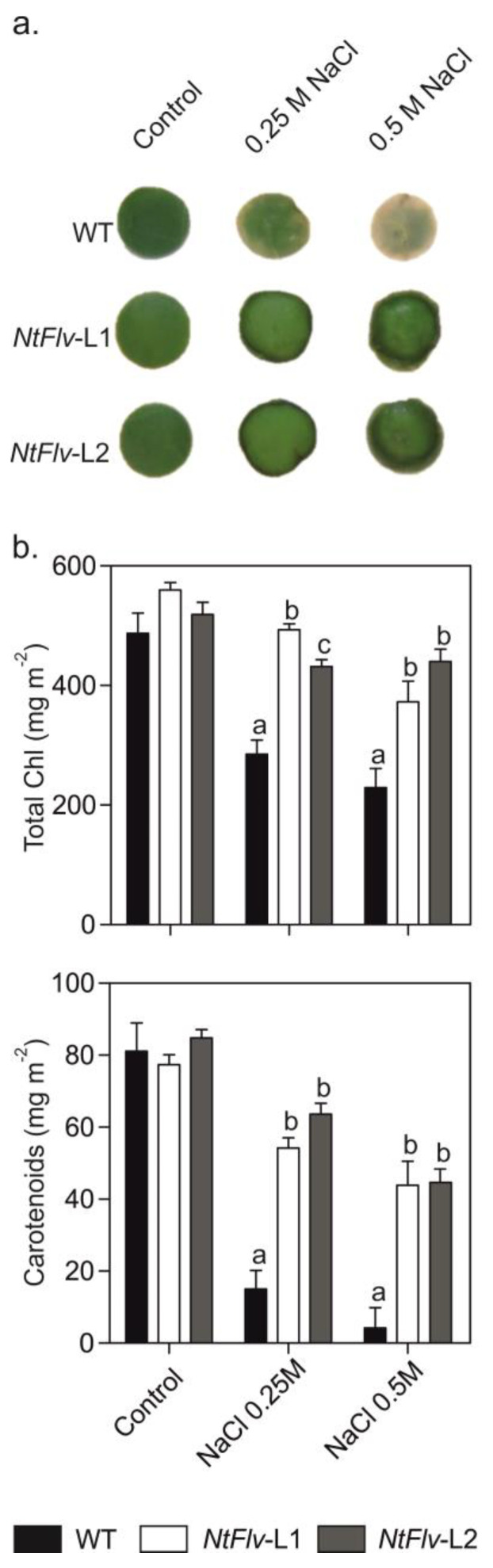
Plastid-located Flv2-Flv4 protected NaCl-exposed leaf tissue from salt toxicity. Leaf discs from six-week-old WT and Flv-expressing tobacco plants were incubated at different NaCl concentrations for five days under growth chamber conditions (**a**). (**b**) Total *Chl* and carotenoids were determined at the end of the treatment using ethanol extraction as described in Section 4.8 of Materials and Methods. Means ± SE of five biological replicates are reported. Different letters indicate significant differences at *p* ≤ 0.05, determined using one-way ANOVA and Tukey’s multiple comparison tests.

If Flv2-Flv4 exerted its protective effect in chloroplasts by relieving the excess of excitation energy and reducing equivalents on the PETC, symptoms of other environmental challenges that cause electron sink limitations could also be ameliorated by the presence of these flavoproteins. Then, the effects of chloroplast Flv2-Flv4 on salt stress tolerance were assayed by incubating tobacco leaf discs from six-week-old plants with 0.25 M and 0.5 M NaCl. As expected, exposure of WT discs to NaCl resulted in leaf tissue bleaching after five days of incubation (Figure 4a), caused by degradation of chlorophylls and carotenoids (Figure 4b). Flv2-Flv4 expression conferred significant protection against salt-dependent pigment deterioration (Figure 4).

Salt stress was also applied to tobacco and Arabidopsis seedlings cultured in 0.8% (*w*/*v*) agar containing half-strength Murashige and Skoog [33] medium (0.5xMS-agar). Figure S6 shows that root elongation of WT tobacco plantlets was progressively arrested as the salt levels were raised, with chloroplast Flv2-Flv4 providing significant protection at moderate NaCl concentrations (75–100 mM). Exposure of Arabidopsis seedlings to 100 mM NaCl yielded similar results, with *AtFlv*-L2 and *AtFlv*-L3 lines displaying the highest levels of tolerance (Figure S7).

**Figure 5:**
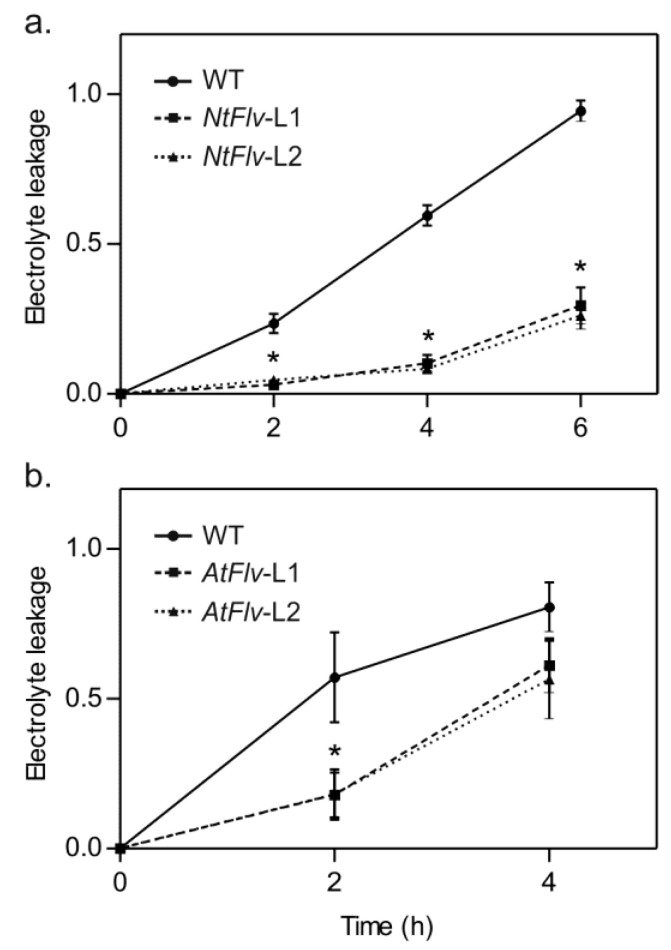
Flv-expressing plants displayed increased tolerance to MV toxicity. Leaf discs were obtained from six-week-old tobacco (**a**) or four-week-old Arabidopsis (**b**) plants and exposed to 5 μM MV as described in Section 4.9 of Materials and Methods. The release of cellular ions into the incubation medium was monitored at the times indicated using a conductivity meter, and the fraction of total electrolytes is represented in the ordinates. The data are the means of six biological replicates ± SE. *: the means differed significantly (*p* ≤ 0.05) from the performance of WT plants using one-way ANOVA and Tukey’s multiple comparison tests.

Chloroplast Flv2-Flv4 also afforded protection to oxidative stress resulting from incubation of illuminated leaf discs with methyl viologen (MV), a redox-cycling herbicide that generates ROS in chloroplasts [34]. Tissue damage was evaluated by measuring ion leakage. As shown in Figure 5a, cell lysis was virtually complete in WT tobacco discs after 6 h of incubation. In contrast, discs of *NtFlv*-L1 and *NtFlv*-L2 plants leaked less than 30% of their electrolyte contents by that time (Figure 5a), indicating that Flv2-Flv4 differentially preserved cell integrity under oxidative stress conditions. A similar protective effect was obtained when assaying Arabidopsis discs, even though the time courses were different, presumably reflecting differences in species or physiological conditions (Figure 5b).

### 2.4. Chloroplast Flv2-Flv4 Enhanced Drought Tolerance in Tobacco and Arabidopsis Plants

**Figure 6:**
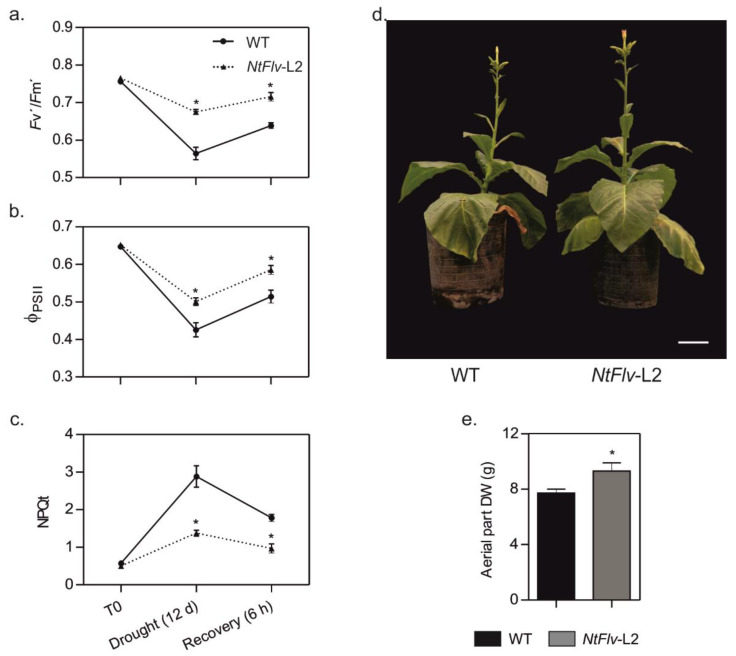
Expression of Flv2-Flv4 in chloroplasts protected photosynthetic activities and improved biomass accumulation in drought-stressed tobacco plants. WT and *NtFlv*-L2 plants were grown in soil for 35 days and subsequently exposed to a water limitation regime as described in Section 4.10 of Materials and Methods. The values are means ± SE of 10 biological replicates. *: the means differed significantly (*p* ≤ 0.05) from the performance of WT plants using one-way ANOVA and Tukey’s multiple comparison tests. Determinations of photosynthetic parameters *Fv’*/*Fm’* (**a**), Φ_PSII_ (**b**), and NPQt (**c**) are described in Section 4.11 of Material and Methods. (**d**,**e**) WT and *NtFlv*-L2 plants grown in soil were exposed to three rounds of water restriction. Typical phenotypes (**d**) and aerial part dry weight (DW) (**e**) of WT and *NtFlv*-L2 plants at the end of the treatment. Bar = 8 cm.

The potential impact of Flv2-Flv4 on drought tolerance was assayed for tobacco and Arabidopsis transformants. Plants from line *NtFlv*-L2, expressing the highest Flv2-Flv4 levels (Figure 1d), were grown in soil for 35 days and then exposed to water deprivation for 12 days, which decreased the field capacity (FC) of the soil to about 30%. Under these conditions, the *Fv’*/*Fm’* parameter (determined in light-adapted leaves; see Section 4.11 in Materials and Methods) dropped from ~0.8 to less than 0.6 in WT tobacco plants, indicating significant damage to PSII (Figure 6a). The presence of Flv2-Flv4 in the transformants largely prevented this decline. In addition, the quantum yield of PSII (Φ_PSII_), which provides an estimation of the electron flow through this photosystem [29], was decreased by the drought regime in both genotypes, but significantly less in the Flv-expressing line (Figure 6b). Instead, the magnitude of the NPQt value, which reflects the dissipation of the excess light energy into various processes by the PETC [35], was increased almost six-fold by water limitation in WT leaves, but only two-fold in the transformant (Figure 6c), suggesting that Flv2-Flv4 could be acting as an extra dissipative mechanism to cope with the over-reduction of the PETC.

After 12 days of drought treatment, plants were re-watered to 70% FC and allowed to recover for 6 h. Photosynthetic parameters improved for both lines, but significant differences still remained between WT and *NtFlv*-L2 plants (Figure 6a–c). Impairment of photosynthetic activities was reflected by protracted growth of WT plants as the treatment was repeated two more times until flowering (~60 days), an effect that was significantly mitigated by Flv2-Flv4 expression in the transformants (Figure 6d,e).

Similar results were obtained with Arabidopsis plants subjected to severe drought conditions. After 10 days of water withdrawal, plants from all genotypes exhibited stress symptoms, but both leaf wilting and chlorosis were largely prevented in the three independent *AtFlv* transformants (typical results shown in Figure S8a). Analysis of photosynthetic activities revealed that Φ_PSII_ values were protected from drought-dependent decline by chloroplast Flv2-Flv4 as in tobacco (Figure S8b). The oxidation state of the plastoquinone pool, as monitored by the qL parameter, was also maintained at significantly higher values in the transgenic lines under water limitation (Figure S8c), confirming a protective role of the Flv2-Flv4 dimer on the photosynthetic machinery in the stressed plants.

### 2.5. Metabolic Profiling in Leaves of Drought-Stressed Arabidopsis Plants Expressing Flv2-Flv4

**Figure 7:**
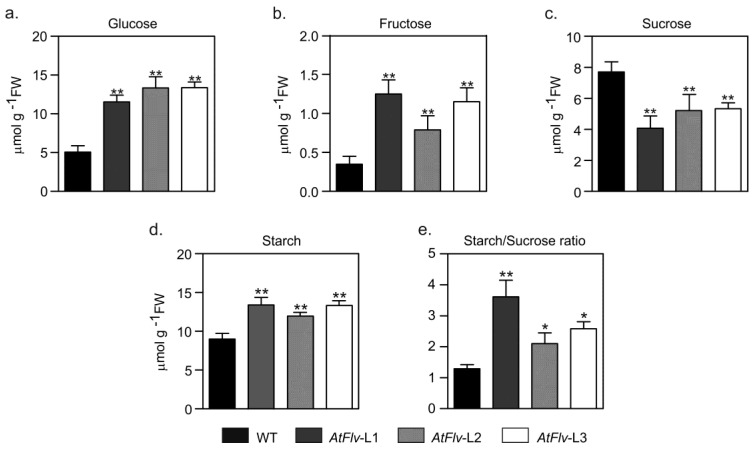
Flv2-Flv4 presence favored a sucrose-to-starch shift in drought-stressed Arabidopsis plants. Extracts were prepared from leaves of four-week-old plants after 10 days of water deprivation, and the levels of glucose (**a**), fructose (**b**), sucrose (**c**), and starch (**d**) were determined as described in Section 4.12 of Materials and Methods. (**e**) Starch/sucrose ratio. Carbohydrate contents are given as means ± SE of seven biological replicates. FW, fresh weight. *, **: the means differed significantly (*p* ≤ 0.05 and *p* ≤ 0.01, respectively) from the performance of WT plants using one-way ANOVA and Tukey’s multiple comparison tests.

When grown under chamber conditions, leaf contents of starch and soluble carbohydrates (sucrose, glucose, and fructose) were slightly higher in Flv-expressing Arabidopsis plants relative to WT siblings, albeit without statistical significance in most cases (Figure S9). Water deprivation led to a major build-up of leaf glucose, especially in the transformants (Figure 7a). Fructose levels were not affected by drought in WT leaves, but rather rose up to three-fold in *AtFlv* counterparts compared to control conditions (Figure 7b). Sucrose contents, instead, increased ~10-fold in non-transformed plants but less than five-fold in Flv-expressing lines after the transition from control to drought conditions (Figure 7c). Finally, starch displayed a moderately higher level in the wild type under water restriction, but almost doubled in the transformants (Figure 7d). As a consequence, Flv2-Flv4 presence caused a major increase in the starch-to-sucrose ratio upon drought stress in L1 and to a lesser extent in L2 and L3 *AtFlv* lines relative to WT values (Figure 7e).

**Figure 8:**
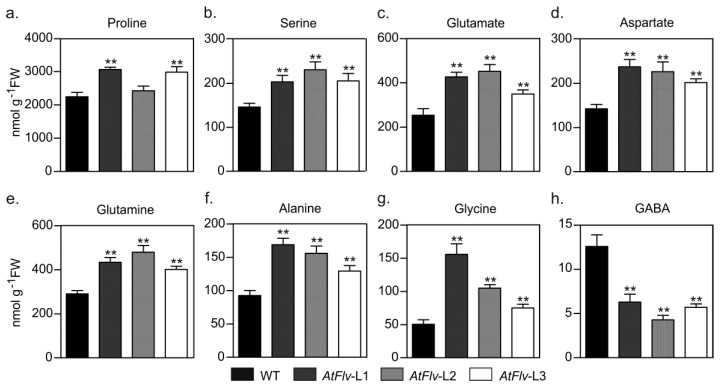
Effect of chloroplast Flv2-Flv4 on the concentrations of free amino acids in Arabidopsis leaves exposed to drought stress: (**a**) proline, (**b**) serine, (**c**) glutamate, (**d**) aspartate, (**e**) glutamine, (**f**) alanine, (**g**) glycine, and (**h**) GABA. Extracts were prepared from leaves of four-week-old plants exposed to 10 days of water deprivation, and amino acid levels were determined as described in Section 4.12 of Materials and Methods. Data shown as means ± SE of seven biological replicates. **: the means differed significantly (*p* ≤ 0.01) from the performance of WT plants using one-way ANOVA and Tukey’s multiple comparison tests.

Most amino acids accumulated to similar levels in leaves from the various lines cultivated under Arabidopsis chamber conditions (Figure S10). Exposure of WT plants to water limitation led to significant increases in several amino acids (Figure 8), most conspicuously the stress marker proline, whose levels were over 40-fold higher in the treated plants compared to watered controls (Figure 8a). With the exception of γ-amino butyric acid (GABA), the presence of plastid-borne Flv2-Flv4 augmented amino acid contents even further (Figure 8). Serine experienced a minor increase upon hydric stress in non-transformed plants, whereas it almost doubled in Flv-expressing siblings (Figure 8b). Accumulation of amino acids involved in N mobilization (glutamate, aspartate, glutamine), as well as alanine and glycine, was significantly higher in the stressed transformants relative to WT counterparts (Figure 8c–g). In contrast, drought caused a major build-up of GABA contents in WT leaves, but not in the transformants (Figure 8h).

## 3. Discussion

Chloroplasts are particularly rich in adaptive responses able to relieve the excess of reducing equivalents and excitation energy on the PETC that occurs under adverse environmental conditions, and to direct them into productive or dissipative pathways. The most recently described of these AET routes is mediated by electron carrier proteins of the FDP family, which are able to accept reducing equivalents from various components of the PETC and deliver them to O_2_ and probably other electron acceptors [7,9,11,12].

Three different FDP systems have been described in photosynthetic organisms. The most extensively studied among them is represented by Flv1-Flv3 and their eukaryotic orthologues FlvA-FlvB, which are found in all phototrophs except angiosperms. Their expression in various plant species revealed that they can functionally interact with the PETC in angiosperm chloroplasts, but their O_2_ photoreduction activity only becomes significant in lines deficient in CET, suggesting that improvements in the efficiency of this latter pathway might explain Flv1-Flv3 disappearance from flowering plants [19,21].

In contrast to Flv1-Flv3 proteins, Flv2-Flv4 distribution is restricted to β-cyanobacteria. Analysis of their expression in response to environmental stimuli offered clues on their putative role. The *flv2*/*4* operon is repressed as CO_2_ levels are increased above the normal atmospheric fraction of 0.04%, and strongly induced by high irradiation [10,24]. Accordingly, *Synechocystis* mutants deficient in *flv2*/*4* genes were hypersensitive to light stress, whereas over-expression of the operon improved growth under high irradiation [10]. Faster re-oxidation of the Q_A_ acceptor of PSII and decreased production of singlet O_2_ in the overexpressing cells indicated that Flv2-Flv4 could act as electron sinks under these stress conditions, dissipating excess electrons and preventing over-reduction of the PETC [10]. The possibility of a similar activity in the chloroplasts of angiosperms was unknown.

To address this question, we introduced Flv2 and Flv4 proteins from *Synechocystis* in tobacco and Arabidopsis chloroplasts (Figure 1 and Figures S1 and S2), and assayed the stress tolerance of the resulting transformants. AET pathways such as those driven by Flv2-Flv4 are usually regarded as “wasteful” because they represent futile redox cycles between water molecules without production of NADPH, and are expected to be employed only as a last resort under stress conditions to avoid even worse consequences for the organism. However, expression of cyanobacterial Flv2-Flv4 in plants did not introduce growth penalties (Figure S3), indicating that they were not affected by the energy investment involved in the expression and function of these FDPs.

Although tobacco plants transformed with the *flv2*/*flv4* genes displayed improved tolerance to high irradiation (Figure 2), *Synechocystis* cells overexpressing the same proteins failed to increase light protection in the absence of Sll0218 [26]. The reasons for this difference are unclear. In cyanobacteria, Sll0218 is involved in PSII stabilization and phycobilisome attachment, thus playing a major role in the preservation of PSII integrity and function under stress [10,23,26]. This protective role of Sll0218 might be more important than the electron sinking activity of the Flv2-Flv4 complex in phycobilisome-containing organisms. However, high light responses are expected to be different in plants, which lack both Sll0218 and phycobilisomes, suggesting that the dissipative role of the introduced Flv2-Flv4 proteins might acquire a greater relevance, not masked by Sll0218 presence. In any event, the protection conferred to photosynthetic components and activities (Figure 2), and the prevention of ROS build-up under high light (Figure 3 and Figure S5), support the notion that Flv2-Flv4 were able to interact with the PETC and relieve its stress-dependent over-reduction by driving novel AET pathways in the chloroplasts of the transformed plants.

Although the growth of *Synechocystis* cells overexpressing the Flv2-Flv4 operon was analyzed under high light [10,26], the response of the transformed cyanobacteria to other sources of stress was not assayed. A prime consequence of adverse environmental conditions was over-reduction of the PETC, and electron sinks may alleviate this condition and improve stress tolerance. Evaluation of Flv-expressing plants exposed to salt, drought, and oxidative treatments confirmed this prediction. Salinity and water deprivation lead to inhibition of the Calvin–Benson cycle and of NADP^+^ regeneration, resulting in over-reduction of the PETC with increased production of singlet O_2_ at PSII and superoxide at PSI [4,36]. Chloroplasts are therefore primary targets of the damage inflicted by these stresses, resulting in pigment degradation (Figure 4), inactivation of photosynthetic activities (Figure 6a–c and Figure S8b,c), and growth arrest (Figure 6d,e and Figures S6, S7, and S8a) in WT tobacco and Arabidopsis plants. Introduction of the Flv2-Flv4 system in chloroplasts of the two species conferred significant protection for all these parameters in the stressed plants (Figure 4 and Figure 6 and Figures S6–S8), indicating that Flv2-Flv4 function as electron sink is conserved across species.

Previous reports have shown that a high level of energy and sugar content is crucial for plants to develop drought stress tolerance, and an active metabolism represents a fundamental resource to overcome this environmental hardship [37,38]. Flv-expressing plants did show major drought-dependent increases in glucose and fructose compared to the wild type (Figure 7). Noteworthy is that there was a significant sucrose shift into starch in *flv* leaves that was not observed in WT counterparts (Figure 7e), implying a higher capability of the transformants for starch biosynthesis and storage in shoots. Functional delivery of assimilates to sink organs for further growth and reproduction under stress is regarded as a key determinant of successful drought tolerance in a number of species ([39] and references therein).

Enhanced amino acid accumulation is a common feature of several stress situations, including water deprivation [39,40]. Flv2-Flv4 expression exacerbated this response for several proteinogenic amino acids, but prevented up-regulation of GABA levels (Figure 8). The consequences of these observations are two-fold. First, the results suggest that Flv-expressing plants can invest part of the increased sugar levels to synthesize some specific amino acids, including N-rich glutamate, aspartate, and glutamine. They normally serve as a key hub in N mobilization to other amino acids, such as the stress marker proline, which is both an osmoprotectant and an antioxidant, and to N-containing protective compounds such as glutathione and polyamines. Then, metabolic profiling indicates that carbohydrate accumulation and amino acid metabolism were enhanced under drought stress by the presence of chloroplast Flv2-Flv4, whereas the GABA shunt pathway was inhibited. This metabolic behavior agrees well with that of drought-tolerant varieties of other species [38,39].

**Figure 9:**
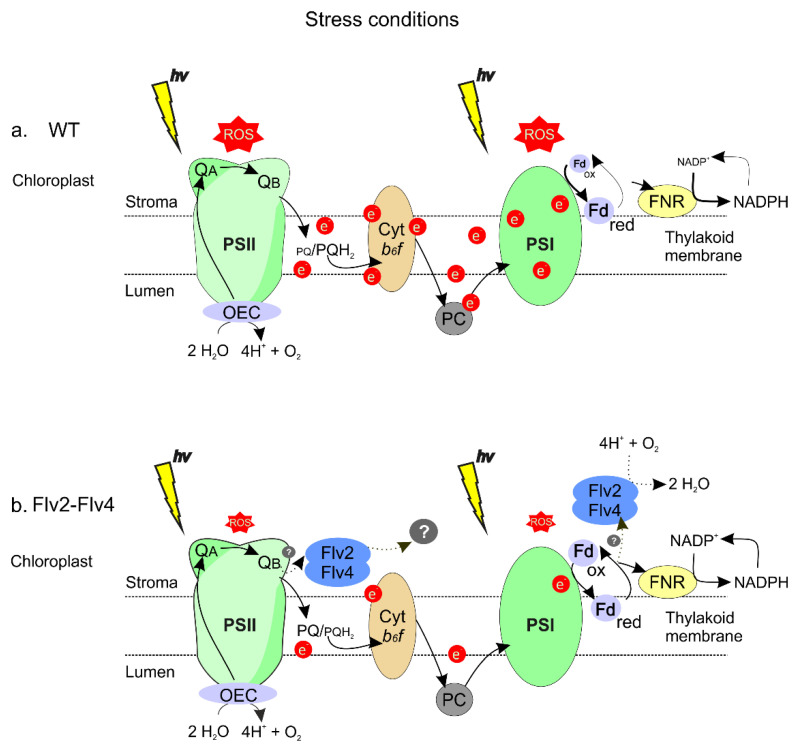
Proposed model for the protective mechanism of Flv2-Flv4 in plastids of transgenic plants. (**a**) Stress conditions in WT plants lead to inhibition of the Calvin–Benson cycle and NADP^+^ regeneration, as well as down-regulation of Fd levels. The photosynthetic electron transport chain (PETC) becomes over-reduced, as indicated by higher fractions of reduced Fd and plastoquinone (PQH_2_). Excess excitation energy (EEE) on the PETC results in ROS build-up in both PSII (mostly singlet oxygen) and PSI (superoxide and peroxides). (**b**) Flv2-Flv4 expression in stressed plants provides an additional electron sink to the PETC, relieving EEE and increasing the oxidation state of chain components. Putative electron donors to the Flv2-Flv4 complex include Q_B_, Fd_red_, and NADPH [10,11]. Adventitious energy and electron transfer to oxygen is attenuated, limiting ROS propagation. OEC, oxygen evolving complex; PC, plastocyanin; Cyt *b*_6_*f*, cytochrome *b*_6_/*f*. Other abbreviations are given in the text.

MV is expected to cause increased superoxide propagation at PSl but not over-reduction of the PETC or singlet O_2_ production. Still, damage caused by this herbicide (i.e., membrane destruction) was significantly protected by Flv2-Flv4 expression (Figure 5). Studies on *Synechocystis* have shown that Flv2-Flv4 can accept electrons from PSII [10,26] and catalyze O_2_ photoreduction under atmospheric (low) CO_2_ levels [11]. However, the two activities apparently result from different electron transfer pathways mediated by the Flv2-Flv4 complex [11]. Oxygen consumption occurs at the reducing side of PSI, similar to Flv1-Flv3, and this activity might explain Flv2-Flv4 protection against MV damage (Figure 5). In contrast, the excess of reducing power at the level of PSII is relieved by an Flv2-Flv4-driven AET from Q_B_ to an unknown final electron acceptor different from O_2_ [10,11]. A similar mechanism involving two alternative electron acceptor sites might be operating in the transgenic plants, and a model illustrating this working hypothesis is provided in Figure 9. Mechanistic details of these two distinct processes have remained elusive, and transgenic plants expressing functional Flv2-Flv4 might represent a valuable tool to address these issues. Results obtained in this research thus prompt for a thorough evaluation of phenotypic performance and photosynthetic activities of Flv2-Flv4-expressing plants cultured under various growth and light regimes.

In conclusion, we report here for the first time the transformation of two species of flowering plants, tobacco and Arabidopsis, with cyanobacterial genes encoding the flavodiiron proteins most restricted in their distribution, and show that they can functionally interact with the chloroplast PETC despite the eons of evolutionary divergence between cyanobacteria and angiosperms. By assaying two plant species we provide evidence supporting the general value of the protective effects described in this article. The broad range of stress tolerance conferred by Flv2-Flv4 when introduced in plants indicates that the use of these flavoproteins might have applications as a biotechnological strategy to improve the field performance of crops grown in suboptimal environments.

## 4. Materials and Methods

### 4.1. Construction of Binary Vectors

Plasmid pCHF3-*TP-flv2-TP-flv4* (Figure 1a), containing the *flv2* (*sll0219*) and *flv4* (*sll0217*) genes from *Synechocystis* sp. PCC6803, each fused in-frame to a 5′-terminal sequence encoding the TP of pea FNR, and placed under the control of individual CaMV-35S promoters, was used to transform both tobacco and Arabidopsis plants. It was constructed in a multiple-step approach using restriction enzymes and the Gibson assembly kit (New England Biolabs, Beverly, MA, USA), according to Gibson et al. [41]. Cyanobacterial *flv* genes were amplified from *Synechocystis* genomic DNA by PCR using high fidelity fusion DNA polymerase (New England Biolabs, Beverly, MA, USA) and oligonucleotides CH2f/CH2rv (*flv2*) and CH4f/CH4rv (*flv4*) listed in Table S1.

The *flv2* and *flv4* coding regions were cloned in the pCHF3-*TP-flv1* plasmid designed by Gómez et al. [20], from which the DNA sequence encoding *flv1* had been excised with *Sal*I and *Sac*I, to generate pCHF3-*TP-flv2* and pCHF3-*TP-flv4*. The region spanning the CaMV-35S promoter, the *TP-flv2* sequences, and the Rubisco *rbcS-E9* terminator were amplified from pCHF3-*TP-flv2* with primers DOB-F and DOB-RV (Table S1), and the amplification product cloned into pCHF3-*TP-flv4* digested with *EcoR*I. Proper orientation and amplification fidelity of the fragments inserted in pCHF3-*TP-flv2-TP-flv4* (Figure 1a) were confirmed by DNA sequencing (UMaine DNA facility, Orono, ME, USA).

To validate chloroplast targeting of the transgenic products, a *GFP*-encoding sequence was fused in frame to the 3′ end of the *flv* transgenes, using pGBW5 Gateway binary vectors driven by the CaMV-35S promoter (Figure S2a) essentially as described by Tula et al. [22].

### 4.2. Plant Transformation and Growth Conditions

Arabidopsis transformation: The *Agrobacterium tumefaciens* strain EHA105 carrying pCHF3-*TP-flv2-TP-flv4* was used to transform *A. thaliana* (Col0) plants in the bolting phase or early flowering by the floral dip method [42]. To obtain homozygous lines, seeds from the T1 generation were grown on 0.5xMS-agar supplemented with 50 mg L^−1^ kanamycin and selected up to the T4 generation. Plants of the various lines were cultured in a soil mixture (70 L substrate 1, 23 L vermiculite, and 372 g plantacote depot 4 m; Klasmann-Deilmann GmbH, Geeste, Emsland, Germany) at 135 µmol photons m^−2^ s^−1^, 8/16-h photoperiod, and 22 °C/18 °C (Arabidopsis chamber conditions). Leaves of 4-week-old plants were used for the experiments, unless otherwise stated.

Tobacco transformation: The pCHF3-*TP-flv2-TP-flv4* plasmid was introduced into the genome of *N. tabacum* (cv Petit Havana) through Agrobacterium-mediated leaf disc transformation [43] using strain GV3101. The selection of transformants and the segregation analysis were also based on kanamycin resistance, and all subsequent experiments were carried out with the T4 homozygous progeny. Seeds were germinated on 0.5xMS-agar, and in the case of transformants, 100 mg L^−1^ kanamycin. After 2 weeks, the seedlings were transferred to soil supplemented with 3 g L^−1^ 15-15-15 (15% N, 15% P, and 15% K), and grown at 200 μmol photons m^−2^ s^−1^, 16/8-h photoperiod, and 25 °C (tobacco chamber conditions). Assays were carried out on the fourth fully expanded leaves of 6-week-old plants, unless otherwise stated.

Transient expression in *N. benthamiana*: The Flv-GFP fusions were transiently expressed in leaves of 6-week-old *N. benthamiana* plants as described by Tula et al. [22], using *A. tumefaciens* strain EHA105 and the pGBW5 vector described above. Leaves sampled 48 h after infiltration were analyzed by CLSM in a Carl Zeiss LSM 880 microscope to monitor GFP fluorescence. Excitation and emission wavelengths were 485 nm and 511 nm, respectively.

### 4.3. RNA Extraction, cDNA Synthesis, and qRT-PCR Reactions

Total RNA was isolated from the leaf tissue of tobacco plants using TriPure reagent (Roche Sigma-Aldrich, St. Louis, MO, USA) according to the manufacturer’s instructions, whereas RNA isolation from Arabidopsis leaves was carried out as described by Logemann et al. [44]. Samples were treated with RNAse-free DNase I (Promega, Madison, WI, USA) prior to cDNA synthesis using oligo d(T)_14_ and M-MLV reverse transcriptase (Invitrogen, Carlsbad, CA, USA) with a template of 1 µg total RNA. The reaction was carried out at 42 °C for 60 min.

The qRT-PCR assays were performed in a Mastercycler_ep realplex thermal cycler (Eppendorf, Hamburg, Germany) using Platinum Taq DNA polymerase (Invitrogen Carlsbad, CA, USA) and SYBR Green I (Roche, Indianapolis, IN, USA) to monitor the synthesis of double-stranded DNA. Relative transcript levels were determined for each sample by the ∆Ct method, since no endogenous *flv* transcripts could be used as WT controls [45]. In the case of tobacco, the primers used were qF2f-Nt/qF2rv-Nt (*flv2*) and qF4f-Nt/qF4rv-Nt (*flv4*), whereas in Arabidopsis, the primers were qF2f-At/qF2rv-At (*flv2*) and qF4f-At/qF4rv-At (*flv4*). Amplification signals were normalized against the levels of *EF1α* (AF120093) cDNA encoding elongation factor 1 in the case of tobacco, and *Ubi10* (At4g05320) cDNA in Arabidopsis. The primers are listed in Table S1.

### 4.4. Western Blot Analysis of Flv-Expressing Tobacco Lines

Total proteins were extracted from tobacco leaf discs, which were homogenized in liquid N_2_ and resuspended at a ratio of 100 mg per 200 µL in urea-containing extraction buffer: 0.2 M Tris-HCl pH 6.8, 3 M urea, 1% (*v*/*v*) glycerol, 8% (*w*/*v*) SDS, 0.5 mM dithiothreitol (DTT), 5% (*v*/*v*) 2-mercaptoethanol, and 1 mM phenylmethylsulfonyl fluoride (PMSF). Samples were incubated at 80 °C for 20 min and centrifuged (18,000× *g* for 15 min at 4 °C). Soluble fractions were transferred to new tubes; mixed with SDS-loading buffer containing 50 mM Tris-HCl pH 6.8, 2% (*w*/*v*) SDS, 1 mM DTT, 10% (*v*/*v*) glycerol, and 0.1% (*w*/*v*) bromophenol blue; and resolved by SDS-PAGE in 12% acrylamide gels. Proteins were electro-transferred to nitrocellulose membranes and probed with polyclonal antisera raised against Flv2 or Flv4 [24]. The membranes were then washed and incubated with 1:10,000 goat anti-rabbit IgG conjugated to horseradish peroxidase (Cell Signaling Tech, Danvers, MA,USA) for 1 h at 22 °C. Blots were carefully washed before incubation with Biolumina detection reagent (Kalium Tech, Buenos Aires, Argentina) as outlined by the manufacturer, and visualized using Bio-Rad Universal Hood II (Hercules, CA, USA). For the determination of Flv2 distribution, tobacco leaves were homogenized in liquid N_2_; resuspended in 50 mM Tris-HCl pH 7.5, 2 mM EDTA, 1 mM MgCl_2_, 1 mM MnCl_2_, 1 mM DTT, and 1 mM PMSF at a ratio of 100 mg per 200 µL; and filtered through 3 layers of miracloth. Filtrates were centrifuged at 18,000× *g* for 10 min at 4 °C. Membrane pellets resuspended in urea-containing extraction buffer and soluble fractions were heated in SDS-loading buffer as described before. After 12% SDS-PAGE, proteins were electro-blotted onto nitrocellulose membranes and probed with the corresponding antisera.

### 4.5. Photoinhibitory Treatment

Tobacco leaf discs (1.2 cm in diameter) were floated with the abaxial side down in 3 mM lincomycin for 12 h in darkness and then exposed to high irradiance stress (1200 µmol photons m^−2^ s^−1^). At the times indicated, the discs were dark-adapted for 30 min and *Fv*/*Fm* was measured with a MultispeQ v1.0 (PhotosynQ Inc., East Lansing, MI, USA).

### 4.6. Thylakoid Isolation and D1 Degradation Assay

Thylakoids from WT and Flv-expressing tobacco plants were isolated according to Camm and Green [46]. Four leaf discs (1.2 cm in diameter, ~100 mg of tissue) were homogenized in 3 mL of chilled buffer containing 50 mM HEPES pH 7.5, 0.4 M sucrose, 2 mM MgCl_2_, 1 mM EDTA, and 0.2% (*w*/*v*) bovine serum albumin with a Polytron homogenizer (Bachofer D-7410, Reutlingen, Germany). The homogenate was filtered through 4 layers of miracloth and centrifuged at 3000× *g* for 5 min at 4 °C. The resulting pellet was resuspended in wash buffer (50 mM HEPES, pH 7.5, 10 mM NaCl) and centrifuged again as above. This second pellet was dissolved in a small volume of wash buffer supplemented with 10% (*v*/*v*) glycerol. *Chl* contents in thylakoid suspensions were estimated after extraction with 80% (*v*/*v*) acetone according to Porra et al. [47].

For the D1 protein assay, thylakoid membrane suspensions were heated in SDS-loading buffer for 5 min at 95 °C and separated by 15% SDS-PAGE. The resolved proteins were transferred to nitrocellulose membranes and detected using a D1 protein-specific polyclonal antibody (Agrisera, Vännäs, Sweden). Quantitative analysis was performed using Fiji software (ImageJ 1.52i, Wayne Rasband, National Institute of Health, USA) [48].

### 4.7. In Situ Detection of Reactive Oxygen Species

ROS were visualized by CLSM in a Carl Zeiss LSM 880 microscope with excitation at 488 nm and emission at 515–530 nm, after leaf staining with the ROS-sensitive fluorescent probe DCFDA, as described by Mayta et al. [49]. Tobacco leaf discs (1.2 cm in diameter) from 4–6 plants of each line were incubated for 12 h with 3 mM lincomycin and then exposed to high light (1200 µmol photons m^−2^ s^−1^) for 2 h. Discs were vacuum-infiltrated with 50 μM DCFDA in 10 mM Tris-HCl pH 7.5, incubated in the dark for 1 h in the same solution, washed briefly, and mounted in water. Images were acquired with a 20× objective (Plan-Apochromat 20×/0.75), image size 512_512 pixels, 16-bit depth. Before recording images, the signal intensity across the entire view was visually inspected in order to prevent signal saturation. Imaging was performed by scanning 26 optical slices (with an interval of 1 mm) of the palisade parenchyma immediately next to the epidermis. Fluorescence intensities were estimated using Fiji software (ImageJ 1.52i, Wayne Rasband, National Institute of Health, USA). Stacks were compiled to single images (z-projections) and presented as a “sum slices” projection type. Fluorescence intensities were calculated using the z-projections.

### 4.8. Salt Stress Treatment

The effect of NaCl was assayed by measuring pigment degradation in tobacco leaf discs, and root elongation in both tobacco and Arabidopsis seedlings. In the first case, discs (1.2 cm in diameter) were floated with the abaxial side down in either distilled water or NaCl in Petri plates and incubated under growth chamber conditions for 5 days. The appearance of chlorotic lesions was monitored daily, and pigments were determined spectrophotometrically after extraction from leaf tissue with 96% (*v*/*v*) ethanol [50].

For the experiments of root elongation, Arabidopsis and tobacco seedlings were grown for 10 days in 0.5xMS-agar until the first two leaves were fully expanded. The seedlings were then transferred to fresh plates containing different concentrations of NaCl and cultured for an additional 7–10 days.

### 4.9. Oxidative Stress Treatment

To evaluate the effects of oxidative stress with the use of the herbicide MV, tobacco discs (1.2 cm in diameter) were incubated with 5 µM MV for 12 h in the dark and then exposed to 800 μmol photons m^−2^ s^−1^ at 25 °C. Arabidopsis conditions were similar, except that discs were 1 cm in diameter and illumination was at 500 μmol photons m^−2^ s^−1^. Cell integrity was evaluated in both cases by measuring ion leakage using a B-173 conductivity meter (Horiba, Kyoto, Japan), as described by Tognetti et al. [51]. At the end of the assay, samples were autoclaved to disrupt all cells, and total electrolyte contents were determined in the resulting solutions.

### 4.10. Drought Stress

Arabidopsis: After 30 days of growth in soil under Arabidopsis chamber conditions, watering was interrupted until FC reached 5%, as monitored with a moisture meter (Delta-T, SM150 kit, Cambridge, UK). Pots were maintained at this FC value for 10 days (severe drought) by adding the required water every alternative day. Leaves of watered and drought-stressed plants were used for determination of photosynthetic parameters and metabolites. Photographs of the plants were taken at the end of the drought treatment.

Tobacco: Plants grown in soil for 35 days under tobacco chamber conditions were exposed to moderate drought stress by withdrawing water until the soil reached 30% FC (12 days without irrigation) followed by a recovery step to 70% FC. Photosynthetic measurements were carried out at the beginning of the treatment, after 12 days of drought and after 6 h of recovery. This treatment (12 days of drought plus recovery) was repeated two more times until flowering. At the end of the assay, aerial parts of the plants were oven-dried for 16 h at 80 °C to determine dry weights.

### 4.11. Photosynthetic Measurements

*Chl* a fluorescence determinations were carried out with a MultispeQ-v1 device controlled by the PhotosynQ platform software [52]. Measurements on tobacco plants were performed at 200 μmol photons m^−2^ s^−1^ red actinic light. The basal fluorescence (*F_0_*) of plants dark-adapted for 30 min was determined using measuring light of 0.02 µmol photons m^−2^ s^−1^, whereas for determinations of minimal fluorescence in the light-adapted state (*F_0′_*), the leaves were transiently subjected to a short exposure (2 s) to weak far-red illumination (730 nm) without background actinic light illumination. Saturating pulses (0.5 s) of 6000 μmol photons m^−2^ s^−1^ were used to obtain maximal fluorescence signals on both dark- and light-adapted states (*Fm* and *Fm’*, respectively). The *Fv’*/*Fm’* parameter (*(Fm’–F_0′_)*/*Fm’*) reflects the maximal efficiency of PSII in light-adapted conditions [29], and was used to follow the photoinhibitory effect of drought during the treatment. The NPQt parameter was determined in light-adapted leaves according to Tietz et al. [35].

In the case of Arabidopsis, photosynthetic parameters were determined after a short photosynthetic induction using red actinic light (135 μmol photon m^−2^ s^−1^) as described by Gómez et al. [20].

### 4.12. Carbohydrate and Amino Acid Profiling

A total of 50 mg aliquots of powdered frozen leaf tissue were extracted in 0.7 mL of 80% (*v*/*v*) ethanol at 80 °C for 1 h. Following centrifugation (19,500× *g* for 10 min), supernatants were evaporated under vacuum at 40 °C, and the residue dissolved in 0.2 mL deionized water. Sugar contents were quantified using the enzymatic method described by Ahkami et al. [53], whereas those of the individual amino acids were obtained according to Mayta et al. [49]. To determine starch contents, ethanol pellets were rinsed twice in 80% (*v*/*v*) ethanol, air-dried at 80 °C for 1 h and resuspended in 0.2 M KOH. The resulting suspension was held at 80 °C for 1 h, adjusted to neutral pH with 1 M acetic acid, and incubated for 12 h at 37 °C in 50 mM NaAc (pH 5.2) containing 7 units mg^−1^ amyloglucosidase. The glucose thereby released was quantified as above.

### 4.13. Statistical Analyses

Results were expressed as means ± SE. Statistical significance was determined by ANOVA for multiple comparison analyses followed by post-hoc Tukey’s HSD test when needed, using the InfoStat program (http://www.infostat.com.ar).

## Supplementary Materials

The following are available online at https://www.mdpi.com/1422-0067/22/3/1178/s1, Figure S1: Flv expression in tobacco plants, Figure S2: Subcellular localization of transgenic Flv2-Flv4, Figure S3: Growth of plants expressing cyanobacterial flv2-flv4 genes, Figure S4: Levels of D1 protein before and after 2 h of high light stress, Figure S5: Expression of plastid-targeted Flv2-Flv4 suppressed ROS build-up in chloroplasts of tobacco leaves exposed to high light, Figure S6: Flv2-Flv4 expression in tobacco chloroplasts improved tolerance to salinity, Figure S7: Flv2-Flv4 expression in Arabidopsis chloroplasts increased tolerance to salinity, Figure S8: Chloroplast Flv2-Flv4 protected photosynthetic activities in drought-stressed Arabidopsis plants, Figure S9: Carbohydrate concentrations in leaves of Flv-expressing Arabidopsis plants grown under chamber conditions, Figure S10: Amino acid contents in leaves of Flv-expressing Arabidopsis plants grown under chamber conditions, Table S1: Oligonucleotides used for PCR and RT-qPCR reactions.

## Abbreviations

PETC	Photosynthetic electron transport chain
ROS	Reactive oxygen species
AET	Alternative electron transport
PS	Photosystem
CET	Cyclic electron transport
NPQ	Non-photochemical quenching
FDPs	Flavodiiron proteins
Fd	Ferredoxin
Q_B_	Quinone-B
WT	Wild type
TP	Transit peptide
FNR	Ferredoxin-NADP^+^ reductase
CaMV-35S	Cauliflower mosaic virus 35S
SDS-PAGE	Sodium dodecyl sulfate polyacrylamide gel electrophoresis
GFP	Green fluorescent protein
Chl	Chlorophyll
CLSM	Confocal laser scanning microscopy
Fv/Fm	Maximum efficiency of PSII
DCFDA	2′,7′-dichlorofluorescein diacetate
AU	Arbitrary units
0.5xMS-agar	Half-strength Murashige and Skoog medium
MV	Methyl viologen
FC	Field capacity
GABA	γ-amino butyric acid
EEE	Excess excitation energy
OEC	Oxygen evolving complex
PC	Plastocyanin
Cyt *b*_6_*f*	Cytochrome b_6_/f
DTT	Dithiothreitol
PMSF	Phenylmethylsulfonyl fluoride
