# Supplementary material for: Expression of Flavodiiron Proteins Flv2-Flv4 in Chloroplasts of Arabidopsis and Tobacco Plants Provides Multiple Stress Tolerance
Source: Int J Mol Sci. 2021 Jan 25;22(3):1178. doi: 10.3390/ijms22031178 (PMC7865949; doi:10.3390/ijms22031178)
Supplement: Supplementary file 1 [file ijms-22-01178-s001.pdf]

Expression of flavodiiron proteins Flv2-Flv4 in chloroplasts of Arabidopsis and tobacco plants provides multiple stress tolerance

Paula Vicino[1#], Julieta Carrillo[1#], Rodrigo Gómez[1,3], Fahimeh Shahinnia[2], Suresh Tula[2], Michael Melzer[2], Twan Rutten[2], Néstor Carrillo[1], Mohammad-Reza Hajirezaei[2]* and Anabella F. Lodeyro[1]*

[1]*Instituto de Biología Molecular y Celular de Rosario (IBR-UNR/CONICET), Facultad de Ciencias Bioquímicas y Farmacéuticas, Universidad Nacional de Rosario (UNR), 2000 Rosario, Argentina.*

[2]*Leibniz Institute of Plant Genetics and Crop Plant Research, OT Gatersleben, Corrensstrasse, D-06466 Stadt Seeland, Germany*

[3]*Present address: Dipartimento di Biotecnologie, Università di Verona, Strada Le Grazie 15, 37134 Verona, Italy.*

***Corresponding authors:**

Mohammad-Reza Hajirezaei, mohammad@ipk-gatersleben.de

Anabella F. Lodeyro, lodeyro@ibr-conicet.gov.ar

#These authors have contributed equally to this work

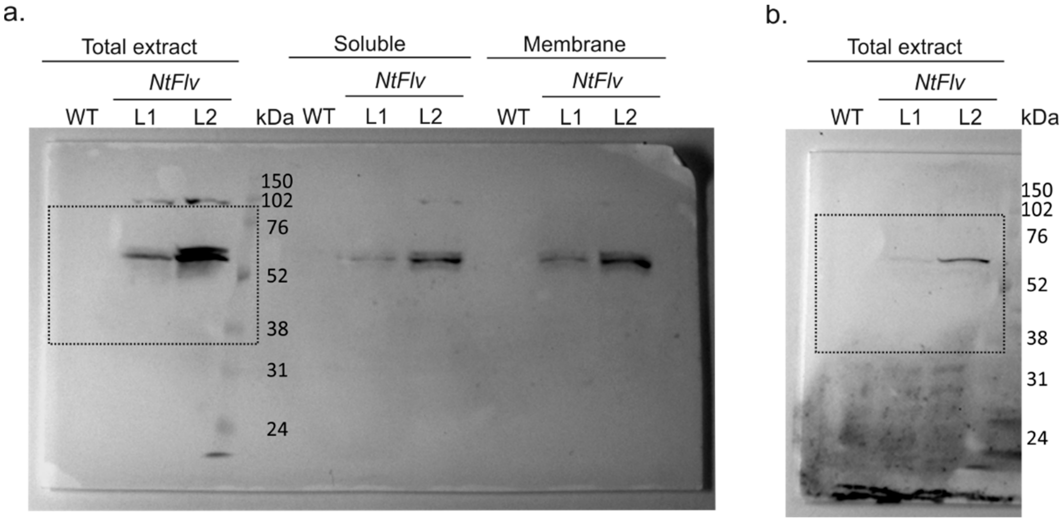

Figure S1. Flv expression in tobacco plants. Distribution of Flv2 in total, soluble and membrane fractions **(a)** and Flv4 in total fraction **(b)** of tobacco leaf extracts. Flv expression of *NtFlv*-L1 and *NtFlv*-L2 tobacco lines was revealed by SDS-PAGE and immunoreaction with Flv2 and Flv4 antisera. The dotted lines indicate the cropped area shown in Fig. 1d. Molecular weight standards are shown in kDa. Uncropped western blot scanned membranes are shown.

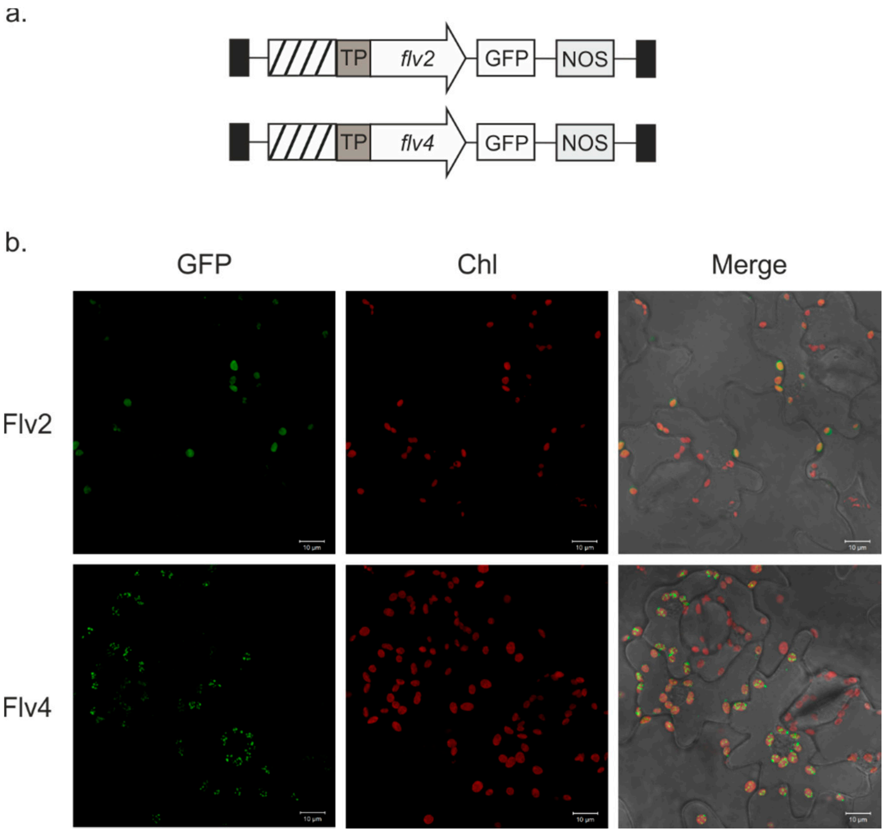

Figure S2. Subcellular localization of transgenic Flv2-Flv4. (a) Schematic representation of the pGBW5 Gateway region containing the pea FNR TP (dark grey boxes) fused to *flv2* and *flv4* (white arrows) and GFP (white boxes) coding sequences. CaMV-35S promoters are shown as striped boxes and NOS terminator sequences as light grey boxes. **(b)** GFP fluorescence in the chloroplasts of *N. benthamiana* transiently transformed with *GFP*-tagged *flv2* and *flv4*. The left panels show GFP fluorescence, the central panels, *Chl* auto-fluorescence and the right panels, merged images with bright field.

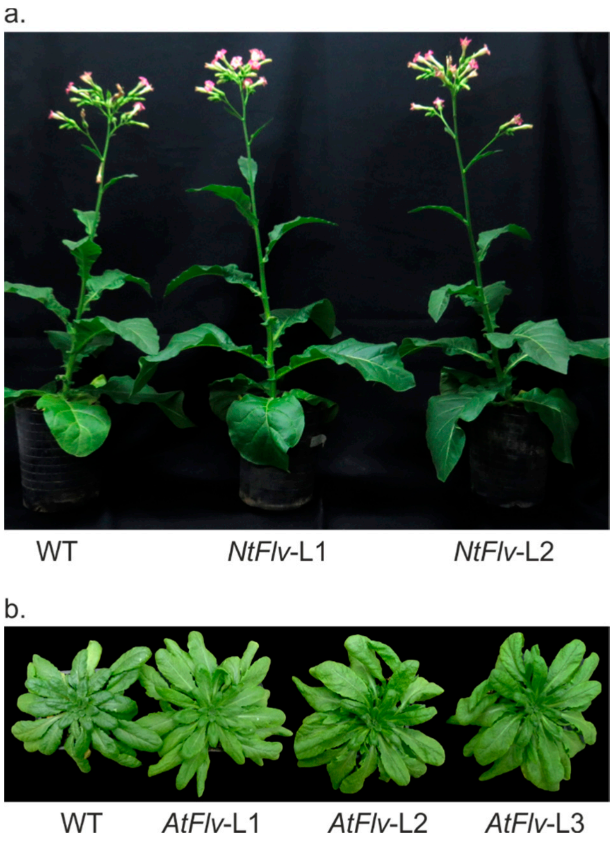

Figure S3. Growth of plants expressing cyanobacterial *flv* genes. Typical phenotypes of 6-weeks old tobacco **(a)** and 4-weeks old Arabidopsis **(b)** plants grown under chamber conditions (see Methods).

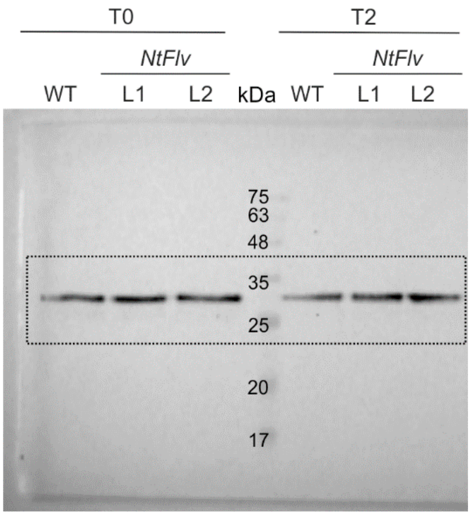

Figure S4. Levels of D1 protein before and after 2 h of high light stress. The uncropped western blot scanned membrane is shown. The dotted lines indicate the cropped area shown in Fig. 2b. Molecular weight standards are shown in kDa.

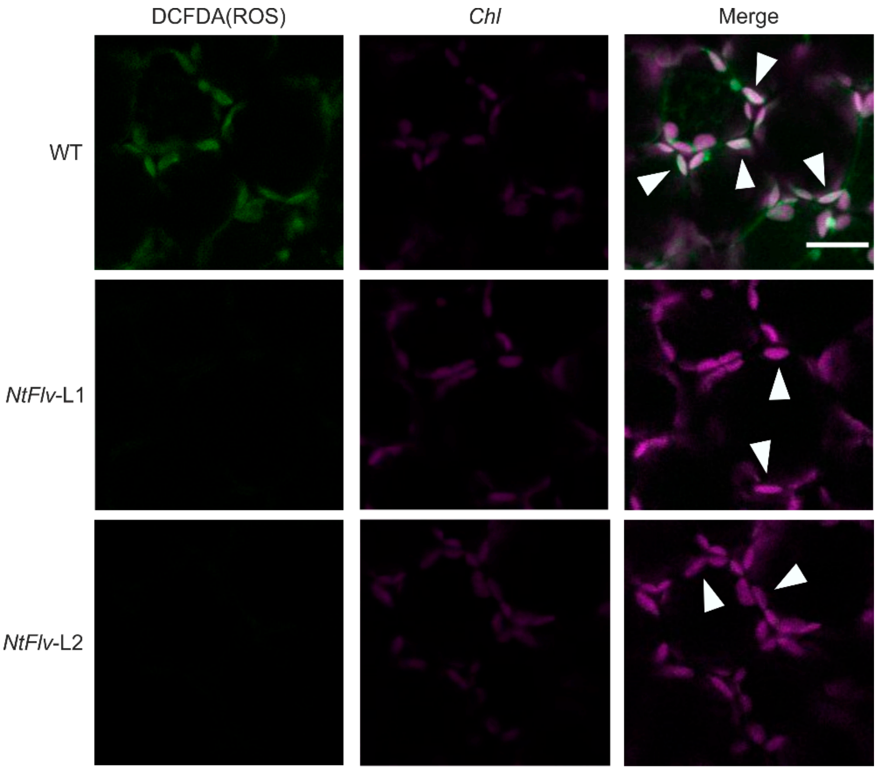

Figure S5. Expression of a plastid-targeted Flv2-Flv4 suppressed ROS build-up in chloroplasts of tobacco leaves exposed to high light. Magnification of leaf tissue stained with the ROS-sensitive probe DCFDA to show single cells of WT, *NtFlv*-L1 and *NtFlv*-L2 genotypes. Bar = 20 μm. ROS (green), *Chl* (magenta) and merge images are depicted. Arrowheads show merge of *Chl* and ROS-derived signals in chloroplasts. All other conditions are those indicated in Figure 3 legend.

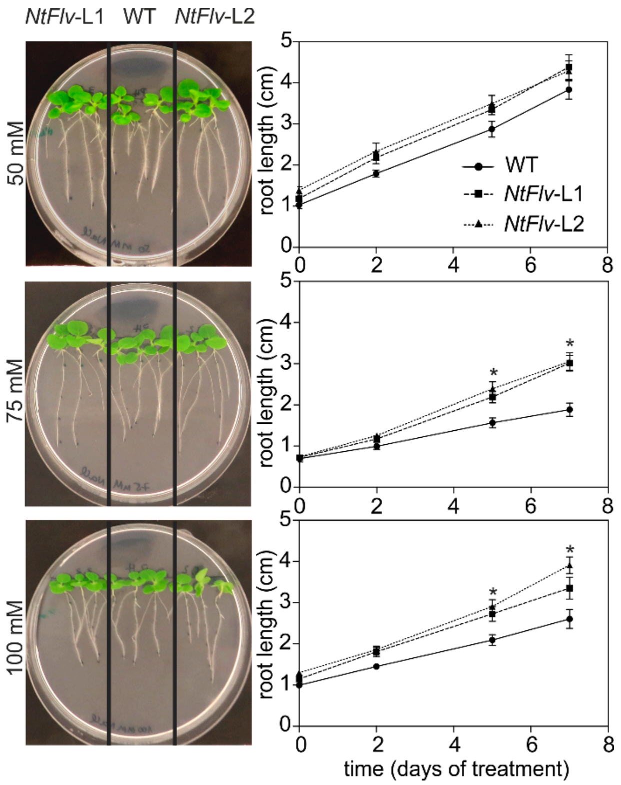

Figure S6. Flv2-Flv4 expression in tobacco chloroplasts improved tolerance to salinity. Plants were grown for 10 days in 0.5xMS-agar and transferred to fresh plates containing the indicated concentrations of NaCl. Photographs were taken after 7 days of treatment. Root lengths were determined in 9 independent specimens, and reported as means ± SE. *: means differed significantly ($P \leq 0.05$) from the performance of WT plants using one-way ANOVA and Tukey's multiple comparison tests. Other details are given in Methods.

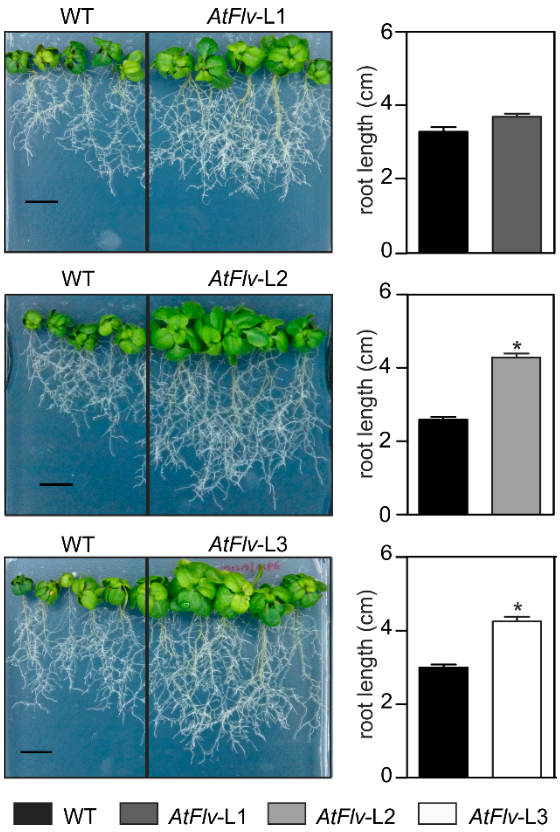

Figure S7. Flv2-Flv4 expression in Arabidopsis chloroplasts increased tolerance to salinity. Plants were grown for 10 days in 0.5xMS-agar and transferred to fresh plates containing 100 mM NaCl. Photographs were taken after 10 days of treatment. Bar = 1 cm. Results are shown as means ± SE (n = 5). Asterisks indicate statistically significant differences compared to the wild type at $P \leq 0.05$, using one-way ANOVA and Tukey's multiple comparison tests. Other details are given in Methods.

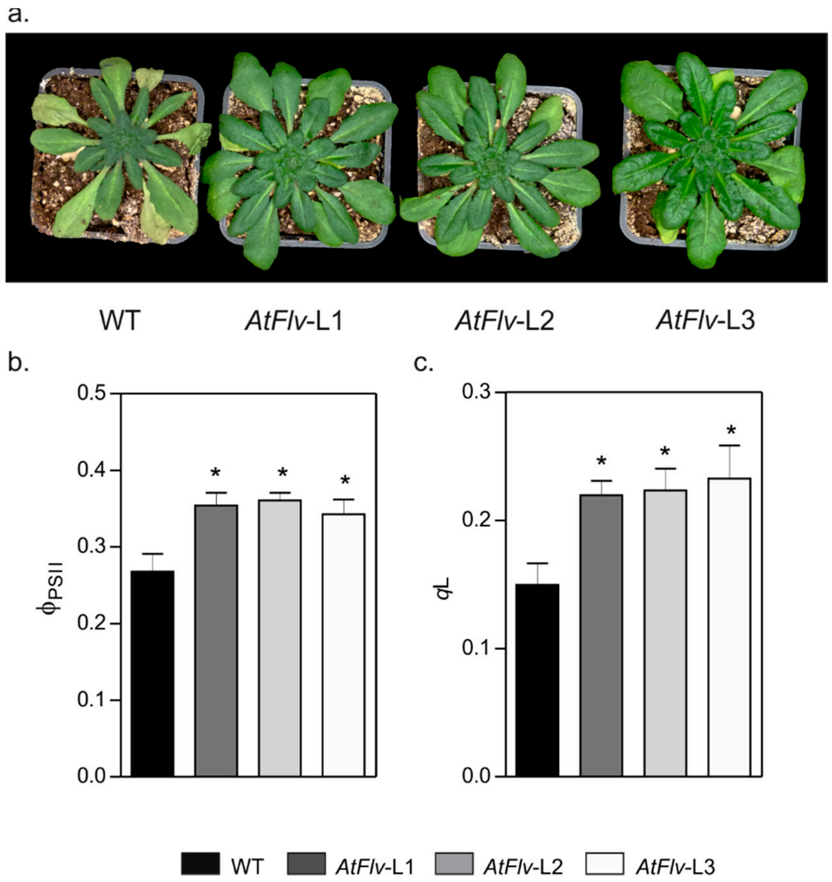

Figure S8. Chloroplast Flv2-Flv4 protected photosynthetic activities in drought-stressed Arabidopsis plants. (a) WT and *AtFlv* plants were photographed after soil was maintained at 5% FC for 10 days, as indicated in Methods.The Φ_{PSII} **(b)** and *q*L **(c)** values of WT and Flv-expressing lines L1, L2 and L3 were determined as described in Materials and Methods. Results are expressed as means ± SE (n = 8). *: means differed significantly ($P \leq 0.05$) from the performance of WT plants.

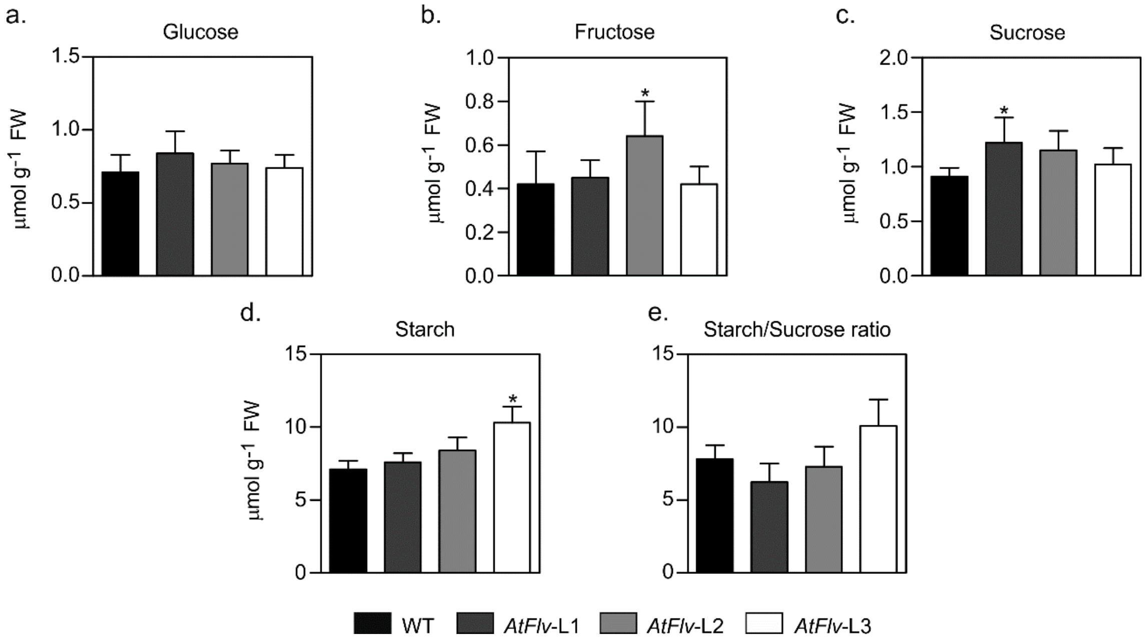

Figure S9. Carbohydrate concentrations in leaves of Flv-expressing Arabidopsis plants grown under chamber conditions. Extracts were prepared from leaves of 4-weeks old plants, and the levels of glucose **(a)**, fructose **(b)**, sucrose **(c)** and starch **(d)** were determined as described in Methods. **(e)** starch/sucrose ratio. Carbohydrate contents are given as means ± SE (n = 8-10). FW, fresh weight. *: means differed significantly ($P \leq 0.05$) from the performance of WT plants using one-way ANOVA and Tukey's multiple comparison tests.

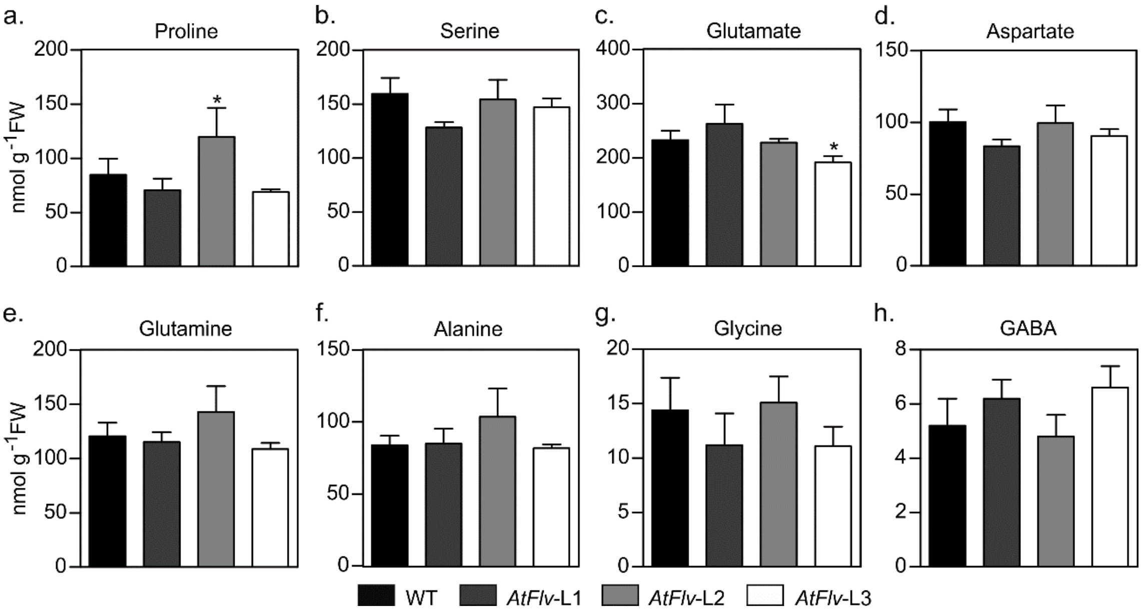

Figure S10. Amino acid contents in leaves of Flv-expressing Arabidopsis plants grown under chamber conditions. **(a)** Proline, **(b)** serine, **(c)** glutamate, **(d)** aspartate, **(e)** glutamine, **(f)** alanine, **(g)** glycine, **(h)** γ-amino butyric acid (GABA). Extracts were prepared from leaves of 4-weeks old plants and amino acid levels were determined as described in Methods. Data shown as means ± SE (n = 7). *: means differed significantly ($P \leq 0.05$) from the performance of WT plants using one-way ANOVA and Tukey's multiple comparison tests.

Table S1. Oligonucleotides used for PCR and RT-qPCR reactions.

CH2f	5′-GGCACCATCAGAGCTATGATTTCTCCAATTGGTGG-3′
CH2rv	5′-CGAAAGCTCTGCAGGCTAATATTGTCCCCCCGATTTG-3′
CH4f	5′-GGCACCATCAGAGCTATGGTTACCCTAATTGATTCTC-3′
CH4rv	5′-CGAAAGCTCTGCAGGTTAGTAGTGGTTGCCCAG-3′
DOB-F	5′-CTATGACCATGATTACGTGAGACTTTTCAACAAAGG-3′
DOB-RV	5′-CTAATCTGGGGACCGTTGATGCATGTTGTCAATC-3′
qF2f-*Nt*	5′-TTATCGGCTCCATTGGTGTGG-3′
qF2rv-*Nt*	5′-AATTGCGAGCATAATGCCAGGG-3′
qF4f-*Nt*	5′-TTTGCTCAAGACCGAACCAACG-3′
qF4rv-*Nt*	5′-CTGGGATTGTTTCTGGGTGAGG-3′
qF2f-*At*	5′-TAGCCAGACCCTCAAAGTAGC-3′
qF2rv-*At*	5′-CAGGGTAGGAGAACCGACAAT-3′
qF4f-*At*	5′-ACCGCTTTGCCGGAGTTA-3′
qF4rv-*At*	5′-GGTGGTGAGTGGCGGTTA-3′
EF1f	5′-TGAGATGCACCACGAAGCTC-3′
EF1rv	5′-CCAACATTGTCACCAGGAAGTG-3′
UBQ10f	5′-CTTCGTCAAGACTTTGACCG-3′
UBQ10rv	5′-CTTCTTAAGCATAACAGAGACGAG-3′
